# The Dementia Diagnostic Process: The Caregiver’s Role and the Challenges Faced

**DOI:** 10.1093/geroni/igaf122.4211

**Published:** 2025-12-31

**Authors:** Lily Gorman, R Amanda Cooper

**Affiliations:** University of Connecticut, Storrs, Connecticut, United States; University of Connecticut, Storrs, Connecticut, United States

## Abstract

The objective of this study was to explore the role of the caregiver when seeking a dementia diagnosis for their spouse, as well as identify the challenges they faced. The process of seeking a dementia diagnosis can be a long and complex task, with numerous barriers along the way. Familial caregivers are deeply involved in this process, yet the role and challenges faced by caregivers are not often the focus of this type of research. Thematic analysis was used to explore caregivers’ experiences of seeking a dementia diagnosis for their spouse. This qualitative study included interviews from 18 caregivers. A number of challenges emerged consisting of both doctor-related barriers: (1) Doctors dismissing or ignoring concerns, (2) Unclear disclosure or miscommunication from doctors, (3) Long, complicated, many step process, (4) Misdiagnosis or mistreatment, (5) Lengthy and frustrating evaluations; and personal or disease-related barriers: (6) Misattribution of symptoms, (7) Fear of hurting their spouse’s feelings, and (8) Denial or hesitancy from their spouse. Seven fluid and dynamic caregiver roles were also identified: Fierce Advocate, The Bad Guy, Determined Investigator, Team Player, Secret Agent, Fearful Fighter, and Pragmatic Realist. These findings provide insight into the complex role caregivers play when seeking a dementia diagnosis for their spouse, and may provide both healthcare professionals and fellow caregivers with an understanding of what this process entails. This study highlights the urgent need to streamline the diagnostic process for people with dementia and their caregivers.

